# The Unexpected Tuners: Are LncRNAs Regulating Host Translation during Infections?

**DOI:** 10.3390/toxins9110357

**Published:** 2017-11-03

**Authors:** Primoz Knap, Toma Tebaldi, Francesca Di Leva, Marta Biagioli, Mauro Dalla Serra, Gabriella Viero

**Affiliations:** 1 Institute of Biophysics, CNR Unit at Trento, Via Sommarive 18, Povo Trento 38123, Italy; primoz.knap@gmail.com (P.K.); mauro.dallaserra@cnr.it (M.D.S.); 2 Yale Cancer Center, Yale University School of Medicine, New Haven, CT 06520, USA; toma.tebaldi@yale.edu; 3 Centre for Integrative Biology, University of Trento, Via Sommarive 9, Povo Trento 38123, Italy; francesca.dileva@unitn.it (F.D.L.); marta.biagioli@unitn.it (M.B.)

**Keywords:** host–pathogen interaction, bacterial toxins, pore-forming toxins (PFTs), long non-coding RNAs (lncRNAs), translation, translational control, ribosome profiling, polysome profiling

## Abstract

Pathogenic bacteria produce powerful virulent factors, such as pore-forming toxins, that promote their survival and cause serious damage to the host. Host cells reply to membrane stresses and ionic imbalance by modifying gene expression at the epigenetic, transcriptional and translational level, to recover from the toxin attack. The fact that the majority of the human transcriptome encodes for non-coding RNAs (ncRNAs) raises the question: do host cells deploy non-coding transcripts to rapidly control the most energy-consuming process in cells—i.e., host translation—to counteract the infection? Here, we discuss the intriguing possibility that membrane-damaging toxins induce, in the host, the expression of toxin-specific long non-coding RNAs (lncRNAs), which act as sponges for other molecules, encoding small peptides or binding target mRNAs to depress their translation efficiency. Unravelling the function of host-produced lncRNAs upon bacterial infection or membrane damage requires an improved understanding of host lncRNA expression patterns, their association with polysomes and their function during this stress. This field of investigation holds a unique opportunity to reveal unpredicted scenarios and novel approaches to counteract antibiotic-resistant infections.

## 1. Membrane Damaging Toxins, Osmotic Imbalance and Translation

According to the World Health Organization (WHO), drug-resistant pathogenic bacteria are estimated to cause 25,000 deaths every year in the European Union alone. These bacteria also lead to high medical costs, prolonged hospital stays and increased mortality [1]. Bacterial pathogens possess a plethora of strategies to subvert host defenses, by secreting biological macromolecules, such as toxins [2,3], which promote bacterial survival within the host environment, for example, by escaping recognition from the immune response. Infections mediated by pathogens often impact the protein synthesis efficiency of the host cell, limiting the production of proteins involved in cellular recovery, like cytokines [4]. Protein synthesis is, in fact, the most energy consuming cellular process, justifying why cells have evolved finely tuned translational control mechanisms to conserve energy and respond quickly to stimuli, if needed [5]. Thus, it is reasonable to presume that translation regulation should be tightly modulated upon bacterial infection through, as yet, poorly understood mechanisms.

One of the most ancient forms of attack exerted by bacterial virulence factors is the formation of proteinaceous pores that cross plasmatic or intracellular membranes [3,6]. These proteins, called Pore-Forming Toxins (PFTs), account for about 25–30% of all bacterial toxins [7]. The intimate relationship between different PFTs and host cell membranes is based on an amazingly large variety of highly specific interactions between toxins and various types of host receptors: sugars, membrane lipids or proteins. While different PFTs use different binding strategies, they all share a common multi-step mechanism of action, for pore formation: (i) release of water-soluble monomers, (ii) binding of monomers to the target membrane, (iii) oligomerization in a non-lytic pre-pore, (iv) insertion of the pore-forming protein portion into the lipid bilayer and opening of nanosized aqueous pores in the host membrane [3,8]. At high toxin doses, this intimate inter-species interaction leads to a massive number of pores, followed by an ionic imbalance [9,10] and indirect or direct membrane damage [8,11]. Cells reply to the osmotic stress by deploying sophisticated mechanisms that counteract the damaging effects of toxins [9,12]. If the activation of host survival or membrane repair mechanisms [10] does not succeed in opposing the stress, cells die, via apoptosis, necrosis or membrane damage. Activation of autophagy and necroptosis have been described as responses to many PFTs, such as aerolysin, vibrio cholerae cytolysin (VCC), *S. aureus* cytolysins [3] and listeriolysin O (LLO) [13]. Even at sub-lytic doses, the binding of toxin monomers or the insertion of a few pores into membranes are still able to provoke extremely diverse cellular responses [11,14]. In fact, the local perturbation of the lipid bilayer upon toxin binding can impact the physiology of the host membrane, by rewiring the physico-chemical organization of the lipid bilayer and altering the proper functionality of host membrane proteins involved in intracellular signaling [15,16].

The proteinaceous pores formed in the host membrane have a wide variety of ionic selectivity and distribution of lumen diameters, ranging from few to tens of nanometers [17]. In any case the pore induces a re-equilibration of ion concentrations across the plasma membrane, resulting in calcium influx and potassium efflux. By a still unclear mechanism, cells are able to detect decreases in the cytosolic potassium concentration, caused by changes in membrane permeability [18]. Calcium is a potent secondary messenger in cells and its ionic imbalance triggers the activation of various signaling cascades to repair the damaged membrane and restore homeostasis. Calcium release from intracellular stores was shown to induce Endoplasmic Reticulum (ER) stress, activating the Unfolded Protein Response (UPR), Ca^2+^ dependent proteases, and Ca^2+^ dependent membrane repair strategies [19]. In addition, the activation of several defense mechanisms, such as MAPK/p38/ERK/JNK, AKT/mTORC pathways [3,18,20] and the inflammasome complex, have been observed [18].

All these events act in concert to control protein synthesis. Potassium efflux induces a transient stop in protein synthesis upon PFT treatment [14,18], a somehow expected outcome since translation can be controlled directly [21] or indirectly through ion fluxes [22]. Moreover, the abovementioned activation of MAPK/p38/ERK/JNK and AKT/mTORC controls the functionality of general translational factors, i.e., eIF4E, eIF2α and eEF2 [5]. Similarly, the crosstalk between potassium efflux and calcium influx can activate the PERK signaling pathway through the UPR, a sensor of ER stress. PERK controls translation via phosphorylation of eIF2α, thereby globally suppressing translation initiation [23]. Overall, the equilibrium between activation and inactivation of translation factors allows cells to enter a low-energy consumption state, in parallel to a rewiring of protein synthesis. Such expedients can facilitate cell survival until recovery of membrane integrity, pointing towards translation as a major hub in promoting cell endurance upon infection and osmotic stress.

Despite this evidence, very few studies have explored the global landscape of changes at the translational (Table 1) or transcriptional [7] levels, occurring as a host response to virulent attacks. Indeed, most of the available studies have focused on transcriptional variations induced by defined immune-stimulatory ligands, such as lipopolysaccharide, with a very recent exception where the host translation response to pathogen infection was monitored by ribosome profiling [24]. Given these still sparse observations, a clear gap of knowledge exists on the precise involvement of translational control in tuning host protein synthesis after exposure to pathogens. This fact preludes a new and interesting field of investigation.

## 2. Host Long Non-Coding RNAs (LncRNAs): An Overlooked Toolkit for Controlling Gene Expression in Host–Pathogen Interaction Studies

Non-coding RNAs (ncRNAs) are very good candidates for the specific and tight regulation of protein synthesis in cells experiencing stresses, such as pore formation and ionic imbalance. Among ncRNAs, long non-coding RNAs (lncRNAs) represent a long-time neglected class of molecules, found in animals and plants. What is striking is that in humans, the number of genes encoding for lncRNAs almost matches the number of protein-coding genes [30]. Importantly, the Encyclopedia of DNA Elements (ENCODE) project, as well as the RIKEN Functional Annotation of the Mammalian Genome (FANTOM 5) consortium, proposed a biochemical function for most lncRNAs. Even if the scientific community is far from being concordant on this matter, with many scientists arguing that the term “functional” is misleading, it is possible that the production of these RNAs represents an ideal playground for evolving new mechanisms to control gene expression across all levels, from transcription to translation.

LncRNAs are a sub-group of non-coding RNAs, loosely defined as transcripts that are longer than 200 nt with no apparent protein coding potential. They can be classified according to two criteria: their genomic position and their mechanism of action or function (Table 2). A significant fraction of lncRNAs appears to be 5′-capped and polyadenylated [31], and presents a similar chromatin arrangement to their actively-transcribed, protein-coding counterparts [32]. However, they do share some common characteristics that distinguish them from *bona fide* protein coding mRNAs (Table 3).

Interestingly, a growing amount of evidence supports the involvement of lncRNAs in regulating post-transcriptional processes and translation [39]. Surprisingly, several lncRNAs have been found to associate with ribosomes [51] and polysomes containing one, two or three ribosomes [52,53]. As to what function they may perform on translation is still a matter of debate. Ribosome profiling experiments have demonstrated that several lncRNAs are in fact engaged by ribosomes as mRNAs [51], raising questions about their classification as non-coding. In accordance with this finding, some lncRNAs were in fact shown to produce short peptides [54] with still unknown functions. Alternative hypotheses to short-peptide production, propose that lncRNAs can rather serve as scaffolds or regulatory platforms, facilitating the recruitment of mRNAs on polysomes. New natural antisense lncRNA classes, that hybridize head-to-head to protein-coding genes, have been described as stimulating cap-independent and cap-dependent translation of target sense mRNAs [55]. These antisense lncRNAs are in fact able to specifically bind to the corresponding sense transcript and, by a still debated mechanism, function as ‘ribosome recruiters’. The “ribosome recruitment” activity of these lncRNAs, implicated in cap-independent translation, resides on embedded repetitive elements (SINEB2 in mouse and FRAM/MIRb in human) [39,40], likely inducing a peculiar RNA structural organization that acts as a scaffold for ribosome engagement to protein-coding transcripts. Importantly, this activity is completely independent of transcriptional effects in the sense transcript [39,40].

It is fair to say that when it comes to the involvement of ncRNAs in host–pathogen interaction studies, the class of lncRNAs has taken the back seat. Most studies on host–pathogen crosstalk are focused on the role played by small ncRNAs, specifically miRNAs [56,57,58,59]. Studies addressing specific lncRNAs are mainly restricted to their involvement in viral infection [43,56,57,58,59]. Indeed, studies considering the role of host lncRNAs transcription during bacterial infection are currently limited to only a handful of examples, nicely reviewed very recently [60]. These pioneering studies shed light on lncRNAs possibly playing an important role in the cell’s response to bacterial infection or in the induction of inflammation, through Toll-like receptor ligands [61].

## 3. Are LncRNAs Overlooked Translation Regulators in Host–Pathogen Crosstalk?

To our knowledge, no information has been collected yet concerning the involvement of lncRNAs in modulating host translation upon either bacterial infection or treatment with virulent factors as PFTs. Indeed, we illustrated that translation is a major hotspot amid the host–pathogen fight for survival. Hence, the impact of PFTs in tuning host protein synthesis efficiency to limit the production of proteins, by triggering the expression and direct recruitment of lncRNAs on ribosomes or polysomes, is likely more than a simple hypothesis.

**Figure 1:**
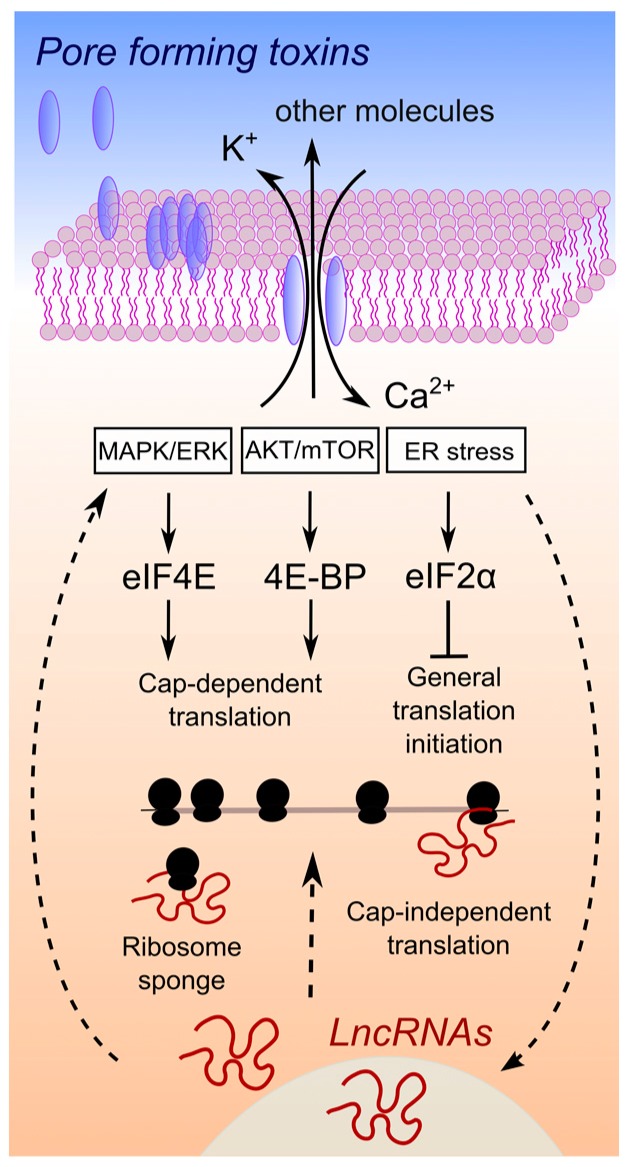
Hypothesis of interplay between lncRNA expression changes and the control of protein synthesis upon pore formation. Upon pore formation, efflux of potassium ions and influx of calcium ions are well known to occur, due to the activity of a large variety of PFTs. A simplified connection between ion imbalance and the activation of three major pathways is depicted (for a complete discussion please refer to the excellent review in [12]). These pathways control downstream target proteins, which are general factors of translation. Straight arrows connect processes related to the activation of pathways that control translation, proven to be involved in the response to ion imbalance triggered by pore-forming toxins or bacterial pathogens. In several cases, an association between lncRNA expression changes and regulation of these pathways has been demonstrated in cancer [63,64,65] or viral infections [66]. The cause and effect relationship of lncRNAs expression and the activation of pathways that control translation is at present not clear, as well as the mechanism of action behind such a connection. Therefore, we used dashed arrows to link lncRNA expression changes to pathways controlling translation, a connection that has been demonstrated for some lncRNAs but not with respect to bacterial infections, ion imbalance or pore formation by bacterial virulent factors.

Interestingly, combined transcriptomics and proteomics have demonstrated that during hyperosmotic stress, yeast is able to adapt by deploying numerous lncRNAs. The transcriptional interplay between stress-activated protein kinases and the induction of a number of non-coding transcripts [42,62], in turn, regulates the transcription of mRNAs coding for downstream factors of the MAPK pathway [62]. Despite the lack of clear mechanisms of action, these lncRNAs have the potential to induce a time-controlled depression of protein synthesis of their target transcripts, helping adaptation to hyperosmotic conditions [62]. Moreover, in higher eukaryotes, evidence accrued over the very last few years has revealed several examples of associations between lncRNA expression and regulation of the MAPK and AKT pathways in cancer [63,64,65] or of the PERK pathway and ER stress in viral infections [66]. Even if the cause and effect relationship between lncRNA expression and modulation of these well-known pathways is not yet clear, it is tempting to speculate that host cells could take advantage of this class of ncRNAs to finely tune translation and cope with the ionic imbalance triggered by PFT attack (Figure 1).

Given these observations, discovering the functions of other infection-induced lncRNAs and determining their mechanism of action will unquestionably expand our knowledge of the host–pathogen crosstalk. Ribosome profiling and polysome profiling experiments, performed in cells treated with pathogenic bacteria, could greatly improve our comprehension of the role of infection-induced lncRNAs in translation control. Comparing the host’s response to invading bacterial strains, either expressing or lacking specific virulent factors, may give valuable insight into their role in the host–pathogen crosstalk, yielding important advances in understanding the interaction between organisms. Moreover, integration of in vivo and in vitro studies, using silencing and in vitro translation systems, can help to address the coding or non-coding functions of several lncRNAs, already found to be up- or down-regulated in cells, upon exposure to virulent factors. Therefore, further research on how cells use lncRNAs to cope with either bacterial infection or the damage caused by PFTs has a huge potential for unveiling, till now unforeseen, scenarios that might shed new light on host–pathogen crosstalk and reveal, as yet unpredicted, approaches to counteract antibiotic-resistant infections.

## Figures and Tables

**Table 1:** Genome-wide translatome/protein synthesis analyses of host response to virulent factors.

Method	System	Reference
Ribosome profiling	macrophages infected with the intracellular bacterial pathogen *Legionella pneumophila*	[24]
Ribosome profiling	macrophages treated with LPS	[25]
Sucrose gradient ultracentrifugation followed by microarray analysis	SH-SY5Y cells treated with lytic and sub-lytic doses of α-haemolysin	[14]
Pulsed SILAC proteomics	dendritic cells treated with LPS	[26]
Sucrose gradient ultracentrifugation followed by PCR array analysis	RAW 264.7 murine macrophages treated with ribotoxic mycotoxin DON	[27]
Sucrose gradient ultracentrifugation followed by microarray analysis	human monocyte-derived dendritic cells treated with LPS	[28]
Sucrose gradient ultracentrifugation followed by microarray analysis	macrophage-like J774.1 cells treated with LPS	[29]

**Table 2:** Classification of lncRNAs according to genomic position or mechanism function.

Genomic Position			Mechanism or Function		
*Name*	*Description*	*Reference*	*Name*	*Description*	*Reference*
Intergenic lncRNAs (lincRNAs)	do not overlap with any part of a protein coding gene and are at least 1 kb distant from it	[33]	Competing endogenous RNAs (ceRNAs)	also called miRNA “sponges”, which participate in a microRNA-dependent crosstalk. These lncRNAs share miRNA response elements (MREs) with some mRNAs, thereby sequestering miRNAs	[34]
*Trans*-Natural Antisense Transcripts (NATs)	antisense lncRNAs acting on mRNAs and complementary to transcripts from remote loci.	[35]	Protein “sponges”	bind regulatory proteins, disabling them from interacting with their potential targets	[36]
*Cis*-Natural Antisense Transcripts (NATs)	antisense lncRNAs acting on mRNAs. These lncRNAs *are transcribed* from the same genomic region as their complementary sense transcript	[35]	Scaffolding lncRNAs	act as a scaffold for multiple chromatin remodelling complexes	[37]
Sense-overlapping or transcribed pseudogene lncRNAs	are considered transcript variants of protein coding mRNAs, and overlap with a protein coding gene on the same DNA strand	[38]	SINEUPs	antisense lncRNAs that stimulate cap-independent translation of target sense mRNAs through the activity of an embedded repetitive element	[39,40]
Intronic lncRNAs	located in the introns of protein coding genes without overlapping with their exons	[41]	Stress-induced lncRNAs (silncRNAs)	Induced upon cell stress, permit a faster recovery of the cell cycle delay caused by stress	[42]
			Modulators of Post Translational Modifications	Act on post-translational modifications of proteins, such as ubiquitination and phosphorylation	[43]

**Table 3:** Characteristics of lncRNAs.

Features	Reference
Lack of a single long open reading frame (ORF) > 300 nt	[44,45]
Low expression levels, compared to mRNAs	[46,47]
Longer but fewer exons than protein-coding genes, with a bias toward two-exons transcripts	[48]
Exons with a significantly lower GC content, compared to protein-coding RNAs	[44]
Paucity or absence of introns	[44]
Enrichments in nucleus	[49]
High degree of tissue specificity	[46,48]
Co-expression with neighboring genes	[46]
Low evolutionary conservation of primary sequence	[50]
